# Clinical Impact of Glucose Levels on Patient Outcome after Transcatheter Aortic Valve Replacement

**DOI:** 10.31083/RCM25336

**Published:** 2025-02-21

**Authors:** Haitham Abu Khadija, Mohammad Alnees, Gera Gandelman, Mahdi Awwad, Tal Schiller, Yazan Hamdan, Omar Ayyad, Alena Kirzhner, Gal Sella, Yazid Kashquosh, Nadin Kakoush, Alex Blatt, Jacob George

**Affiliations:** ^1^Department of Cardiology, Kaplan Medical Center and Faculty of Medicine, Hebrew University of Jerusalem, 9160401 Jerusalem, Israel; ^2^Harvard Medical School, Postgraduate Medical Education, Global Clinical Scholer Research Training Program, Boston, MA 02115, USA; ^3^Department of Diabetes, Endocrinology and Metabolism, Kaplan Medical Center and Faculty of Medicine, Hebrew University of Jerusalem, 9160401 Jerusalem, Israel; ^4^Department of Internal Medicine A, Kaplan Medical Center and Faculty of Medicine, Hebrew University of Jerusalem, 9160401 Jerusalem, Israel

**Keywords:** TAVR, diabetes mellitus, aortic stenosis, blood glucose

## Abstract

**Background:**

Limited data are available for evaluating the effect of blood glucose on transcatheter aortic valve replacement (TAVR) outcomes in patients with diabetes. We aimed to assess the impact of glucose levels on short-term and long-term adverse outcomes in patients undergoing TAVR.

**Methods and Results:**

Data from severe aortic stenosis (AS) patients who underwent TAVR from 2010 to 2022 were collected retrospectively. In total, 615 patients were enrolled in the study: Among the total patient population, 43% had diabetes mellitus (DM), with a mean hemoglobin A1c (HbA1c) level of 7.4 ± 2.5. Within this cohort, 33% were classified as having uncontrolled diabetes, while 17% were considered well-controlled. Diabetic patients were younger (80.7 ± 6.8 vs. 82.0 ± 6.8 years, *p* = 0.001) and had more cardiovascular risk factors. No significant differences were found in outcomes between the two groups during the twelve-year follow-up. A multivariable logistic regression analysis was conducted on 270 DM patients to examine the impact of blood glucose levels and HbA1c on outcomes such as arrhythmia, stroke, and acute kidney injury (AKI). For arrhythmia, the odds ratio for HbA1c and blood glucose were 1.1039 (*p* = 0.23), and 0.998 (*p* = 0.76), indicating no significant associations. In stroke cases, HbA1c had an odds ratio of 1.194 (*p* = 0.36), while an odds ratio of 1.020 (*p* = 0.013) for blood glucose indicated a significant association. Notably, for AKI, the odds ratio for HbA1c was 2.304 (*p* = 0.02), indicating a significant link between higher HbA1c levels and increased AKI risk, with blood glucose levels trending toward significance (odds ratio = 1.0137, *p* = 0.061).

**Conclusions:**

Diabetic status is a predictor of short-term outcomes following TAVR. Thus, these screening parameters should be included in risk assessment tools for TAVR candidates.

## 1. Introduction 

Aortic stenosis (AS) is the most common ventricular heart disease [1], particularly in the elderly population and especially in individuals over 80 years. AS is characterized by progressive aortic valve dysfunction, leading to serious health consequences. Timely intervention is crucial, as untreated, severe AS can result in high mortality rates once patients become symptomatic [1, 2].

Transcatheter aortic valve replacement (TAVR) has emerged as the preferred treatment for severe symptomatic AS in patients at high surgical risk [1]. TAVR has resulted in lower morbidity and mortality compared to traditional surgical aortic valve replacement (AVR) in these high-risk patients. The procedure has evolved significantly, with advancements in valve design and imaging techniques enhancing its effectiveness [3].

Diabetes mellitus (DM) is a critical risk factor for both the development and progression of AS, contributing to adverse clinical outcomes in patients undergoing TAVR. Patients with diabetes often experience worse ventricular remodeling and increased complications [4, 5]. However, the impact of DM on TAVR outcomes remains unclear, with studies showing inconsistent findings regarding peri-procedural complications and mortality rates [6, 7, 8].

The relationship between DM and TAVR is still not fully understood, especially the short-term complications in diabetic patients undergoing TAVR due to inconsistent findings about the peri-procedural complications. Our study aims to compare diabetic and nondiabetic patients undergoing TAVR and to examine the effects of periprocedural blood glucose levels on clinical outcomes in patients undergoing TAVR.

## 2. Materials and Methods

### 2.1 Study Design and Population

This retrospective cohort study included all patients with severe symptomatic AS who underwent TAVR between February 2010 and July 2022 at the Kaplan Medical Center, Rehovot, Israel. Patients were categorized into two groups, those with or without DM.

### 2.2 Exclusion and Inclusion Criteria

Inclusion Criteria: Patients aged 18 years and older who underwent TAVR for severe AS were included in the study. Eligible patients receiving self-expandable valve (SEV) or balloon-expandable valve (BEV), had complete periprocedural data and follow-up data.

Exclusion Criteria: We excluded patients without available blood tests, those who experienced periprocedural death (up to 24 hours post-TAVR), and individuals who underwent non-transfemoral access TAVR. Additionally, we excluded 12 patients who had other types of valves implanted. Other exclusion criteria included patients with medical conditions that could affect blood glucose levels, such as malignancies, concurrent infections, chronic inflammatory diseases, and those receiving glucocorticoid therapy within three months before admission.

### 2.3 Data Collection

The data were collected retrospectively from the hospital database, which included the baseline characteristics, lab tests, procedural data, and clinical outcomes, and are illustrated in Tables 1,2 below.

**Table 1.** **Baseline characteristics of patients**.

		Overall	No DM	DM	*p* value
		n = 615	n = 345	n = 270	
Clinical characteristics					
	Age (years)	81.48 ± 7.36	82.05 ± 6.87	80.76 ± 6.87	0.001
	Gender women (%)	54.6%	55.4%	53.5%	0.651
	Body mass index (kg/m^2^)	28.14 ± 4.96	27.52 ± 4.74	29 ± 5.15	<0.001
	Hypertension	90.7%	89.2%	92.6%	0.149
	Dyslipidemia	79.1%	75.7%	83.3%	0.022
	Smoker	11.1%	9.0%	13.7%	0.064
	Atrial fibrillation	30.0%	30.2%	29.7%	0.907
	Coronary artery disease	44.2%	46.2%	41.6%	0.254
	Peripheral vascular disease	16.9%	14.4%	20.1%	0.059
	Past myocardial infarction	16.0%	15.0%	17.4%	0.412
	Past stroke	9.2%	8.2%	10.4%	0.337
	Past CABG	8.2%	5.4%	11.5%	0.017
	STS score	7.93 ± 1.6	7.83 ± 1.72	8.04 ± 1.56	0.517
Laboratory					
	White blood cells (K/uL)	7.47 ± 2.41	7.35 ± 2.57	7.64 ± 2.19	0.028
	Neutrophil-absolute (K/uL)	5.04 ± 2.14	4.91 ± 2.29	5.20 ± 1.93	0.063
	Lymphocytes-absolute (K/uL)	1.62 ± 1.07	1.61 ± 0.96	1.62 ± 1.20	0.600
	NLR	3.80 ± 2.75	3.67 ± 2.67	3.98 ± 2.87	0.157
	Platelets (K/uL)	212 ± 82	210 ± 81	215 ± 82	0.119
	Total cholesterol (mg/dL)	158 ± 40	162 ± 39	153 ± 41	0.003
	Total protein (g/dL)	6.8 ± 0.67	6.8 ± 0.72	6.8 ± 0.59	0.976
	Albumin (g/dL)	3.9 ± 0.38	3.9 ± 0.40	3.9 ± 0.34	0.984
	Creatinine (mg/dL)	1.22 ± 0.91	1.15 ± 0.90	1.31 ± 0.93	0.001
	eGFR (mL/min/1.73 m^2^)	58.12 ± 21.3	64.10 ± 17.2	52.42 ± 24.3	0.012
Echocardiography					
	Mean LVEF (%)	52.8 ± 9.4	53.0 ± 9.4	52.7 ± 9.3	0.633
	Septum thickness (mm)	13.8 ± 3.18	13.6 ± 2.75	14.0 ± 3.68	0.251
	Aortic valve area (cm^2^)	0.75 ± 0.93	0.71 ± 0.51	0.81 ± 1.29	0.033
	Aortic valve gradient (mm Hg)	47.1 ± 16.3	48.61 ± 17.13	45.13 ± 14.89	0.059
Procedure related					
	Self-expandable valve	365 (61.1%)	210 (62.9%)	155 (58.9%)	0.403
	Time (minutes)	86 ± 33	87 ± 34	84 ± 31	0.556
	Contrast (milliliter, mL)	121 ± 58	123 ± 58	119 ± 57	0.461

Values are mean ± standard deviation or n (%). *p* value considered positive <0.05. eGFR, estimated glomerular filtration rate; LVEF, left ventricular ejection fraction; CABG, coronary artery bypass grafting; DM, diabetes mellitus; STS, Society of Thoracic Surgeons; NLR, neutrophil to lymphocyte ratio.

**Table 2.** **Clinical outcomes post-TAVR in diabetic and non-diabetic patients**.

	Overall	No DM	DM	*p*-value
Mortality 24 hours to 30 days	29 (10.9%)	18 (12.7%)	11 (8.8%)	0.310
Mortality within 1 year	48 (7.8%)	20 (5.8%)	28 (10.4%)	0.036
MI	10 (1.7%)	7 (2.1%)	3 (1.2%)	0.374
Bleeding	56 (9.1%)	32 (9.3%)	24 (8.9%)	0.869
Major vascular complication	63 (10.2%)	37 (10.7%)	26 (9.6%)	0.657
Stroke	27 (4.4%)	14 (4.1%)	13 (4.8%)	0.649
AKI	47 (8.0%)	22 (6.7%)	25 (9.7%)	0.175
Arrhythmia	170 (28.7%)	100 (30.2%)	70 (26.7%)	0.350

Data are presented as numbers (%), and the *p* value is considered positive <0.05. AKI, acute kidney injury; MI, myocardial infarction; DM, diabetes mellitus; TAVR, transcatheter aortic valve replacement.

### 2.4 TAVR Procedure and Periprocedural Management

The decisions regarding TAVR vs. AVR, valve type and size, and access were made by a multidisciplinary group consisting of an interventional cardiologist, a cardiac surgeon, an echocardiographic specialist, and a radiologist. The decision to perform a pre-TAVR balloon aortic valvuloplasty was left to the interventional cardiologist at the start of the procedure. The procedures were performed in a hybrid room with a cardiac surgeon on standby. Before undergoing TAVR, all patients received a standard coronary preoperative evaluation and provided informed consent for the procedure and the data collection and analysis.

The SEV-treated patients were implanted with the following valves: corevalve, evolute-R, or evolute-PRO (Medtronic, Inc., Minneapolis, MN, USA). BEV-treated patients were implanted with the following valves: Sapien, Sapien XT, or S3 (Edwards Lifesciences, Irvine, CA, USA). The percutaneous approach and the safety wire technique, along with the Prostar XL vascular closure device (Abbott Vascular, Redwood City, CA, USA), were used for trans-femoral artery access and closure. We considered the procedure duration to be “skin to skin”. The start time was when arterial access was obtained, and the end time was when this access was closed. The standard approach was local anesthesia with conscious sedation. After inserting the femoral sheet, we used heparin to maintain a minimum active clotting time (ACT) of over 250 seconds. If needed, a protamine-heparin antagonist (1 mg for each 100 U of heparin) was administered at the time of vascular closure.

### 2.5 Blood Glucose Levels, Lab Tests, Inflammatory Markers, and Event Definition Criteria

Blood samples were obtained using a sterile syringe without stasis. Laboratory analyses were done pre-procedure, daily post-procedural during the intensive care unit (ICU) stay, and during the physician’s visit to the cardiology ward. The blood test data were retrospectively collected.

Fasting pre-procedural blood glucose (BG) levels were obtained from the blood tests drawn on the morning of the procedure.

The standard follow-up visits were 30 days and six months after being discharged from the hospital. All follow-ups were performed at our medical center. The post-procedural events were defined using the criteria from the Valve Academic Research Consortium 3 (VARC-3). Data on mortality were collected from medical records after a 12-year follow-up period.

### 2.6 Sample Size Calculation

We used a two-sample means independent t-test to determine the sample size required for our study comparing controlled and uncontrolled hyperglycemic states in TAVR patients. Our study aims to compare diabetic and nondiabetic patients undergoing TAVR and to examine the impact of periprocedural blood glucose levels on clinical outcomes. Our sample size calculations were based on parameters estimated from previous studies that reported mean creatinine values and variances [9, 10]. This analysis indicated that a total sample size of 39 participants is needed to achieve a power of 0.80 at a significance level of 0.05. Specifically, this includes 13 participants in the hemoglobin A1c (HbA1c) <5.7 group and 26 participants in the HbA1c ≥7 group, maintaining a sample size ratio of 2:1.

The expected effect size of –0.33 suggests a moderate difference in means between the groups, with the HbA1c <5.7 group having a mean creatinine of 0.82 and the HbA1c ≥7 group having a mean creatinine of 1.15. Additionally, the standard deviations for the HbA1c <5.7 group and the HbA1c ≥7 group are 0.19 and 0.37, respectively, reflecting the unequal variances that will be addressed using Satterthwaite’s *t*-test.

This sample size will ensure adequate power to detect differences in outcomes as we compare diabetic and nondiabetic patients undergoing TAVR while also examining the impact of periprocedural blood glucose levels on clinical outcomes.

### 2.7 Statistical Analysis

The categorical variables are presented as frequencies and percentages, and continuous-type variables are presented as mean ± standard deviation (SD). We tested normality between the various study groups’ continuous variables using a Shapiro-Wilk test. A Mann-Whitney non-parametric test was performed when abnormal distributions were found. When appropriate, we performed a Pearson’s chi-square test for categorical variables. Main effect estimates are presented with their 95% confidence intervals.

**Fig. 1.**
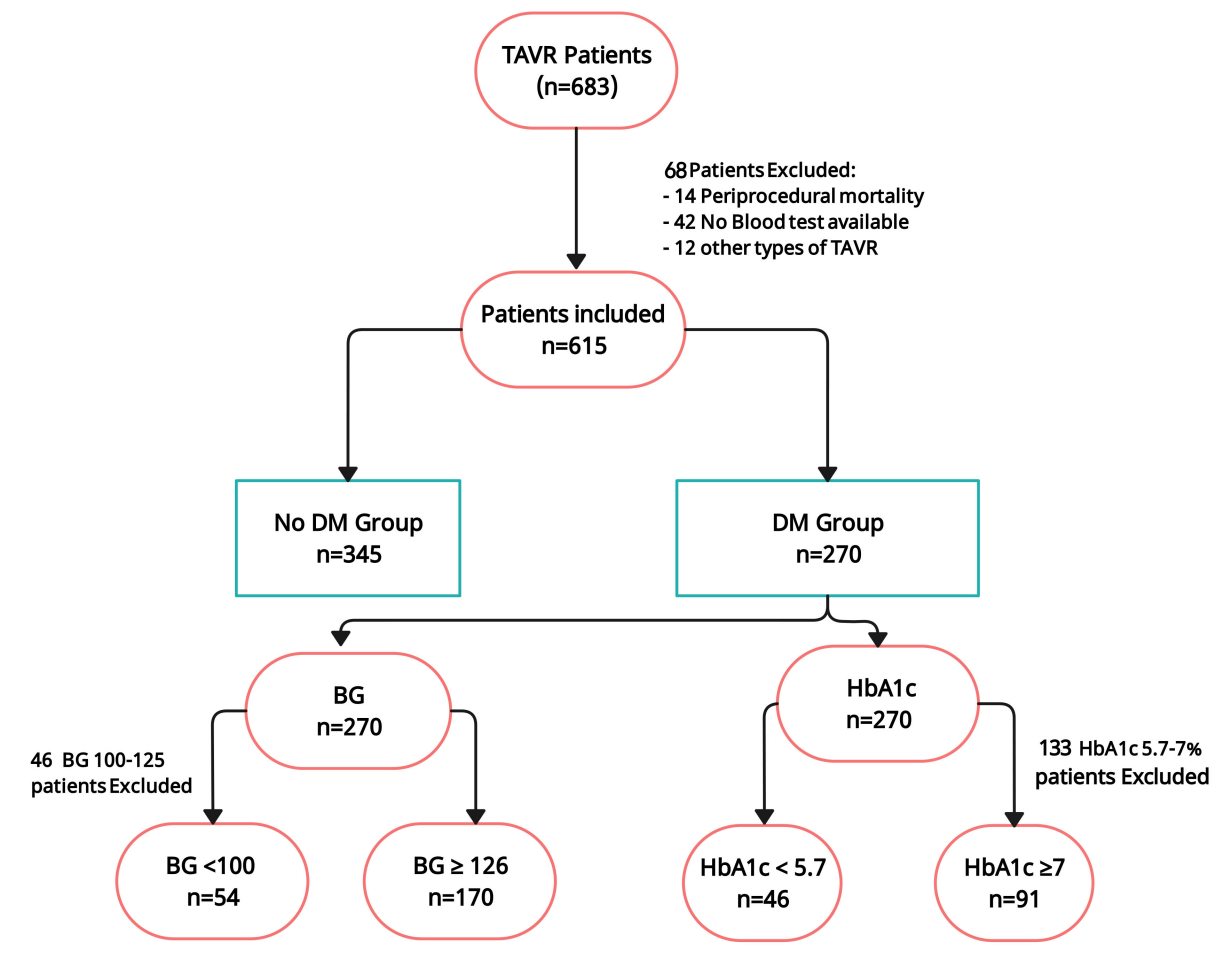
**Study flowchart of patients from 2010 to 2022**. A total of 683 patients were treated with TAVR. After excluding 68 patients, a total of 615 patients were finally included in the analysis. BG, blood glucose; DM, diabetes mellitus; TAVR, trans-aortic valve replacement; HbA1c, hemoglobin A1c.

We divided the patients into two groups; patients with DM were identified based on whether the patient had this diagnosis in previous records or was on prescribed antidiabetic treatment (oral medications or insulin). The other group was patients free of DM. We further subdivided the DM patients into several groups according to pre-procedural BG levels (BG <100 mg/dL, BG 101–125 mg/dL, and BG ≥126 mg/dL). These values were determined based on the universally accepted definition of hyperglycemia in clinical trials and according to baseline HbA1c levels (HbA1c <5.7 and HbA1c ≥7), which indicate controlled and uncontrolled hyperglycemic states respectively (Fig. 1) [11, 12, 13, 14, 15, 16].

We used the Kaplan Meier test for post-six-month cumulative survival analysis to evaluate the survival between the groups. A log-rank test was used when appropriate. *p* values < 0.05 were defined as statistically significant. We use logistic regression multivariable analysis to adjust for confounding factors.

All statistical analyses were done using the SPSS program (Statistical Package for the Social Sciences) (IBM, version 27.0) (IBM Corp., Armonk, NY, USA). This study was approved by Kaplan Medical Center’s local Helsinki Committee.

## 3. Results

### 3.1 Patient and Procedural Characteristics

Fig. 1 shows the study’s flowchart. Six hundred and eighty-three patients were identified for the twelve-year study period. Sixty-eight participants were excluded from the study. The analyzed population included 615 patients (46% male, mean age 81.4 ± 7.3 years) with symptomatic severe AS (mean trans-aortic pressure gradient 47.1 ± 16.3 mmHg) and prohibitive or high operative risk (society of thoracic surgeons (STS) score of 7.93 ± 1.6). The baseline, procedural aspects of the study population, and the two subgroups are summarized in Table 1. Diabetic patients were younger (80.7 ± 6.8 vs. 82.0 ± 6.8 years, *p* = 0.001) and had a higher body mass index (29 ± 5.1 vs. 27.5 ± 4.7 kg/m^2^, *p* < 0.001) than non-diabetic patients. DM patients had more cardiovascular risk factors, including peripheral vascular disease (20.1% vs. 14.4% in non-diabetics, *p* = 0.059), previous coronary bypass surgery (11.5% vs. 5.4%, *p* = 0.017), and higher cholesterol levels (153 ± 41 mg/dL vs. 162 ± 39 mg/dL in non-diabetics, *p* = 0.003). There was no significant difference in blood pressure between the two groups. Patients with DM had a higher serum creatinine level of 1.31 ± 0.93 mg/dL compared to 1.15 ± 0.90 mg/dL in those without DM (*p* = 0.001). Additionally, the estimated glomerular filtration rate (e-GFR) was lower in the DM group at 52.42 ± 24.3 mL/min/1.73 m^2^ versus 64.10 ± 17.2 mL/min/1.73 m^2^ for non-DM patients (*p* = 0.012).

### 3.2 Clinical Outcome

The risk of 30-day mortality (24 hours to 30 days) was identical among patients with and without DM (8.8% for DM versus 12.7% for no DM, *p* = 0.310; Table 2). Patients with DM exhibited a higher one-year mortality rate (10.4% vs. 5.8%; *p* = 0.036).

**Fig. 2.**
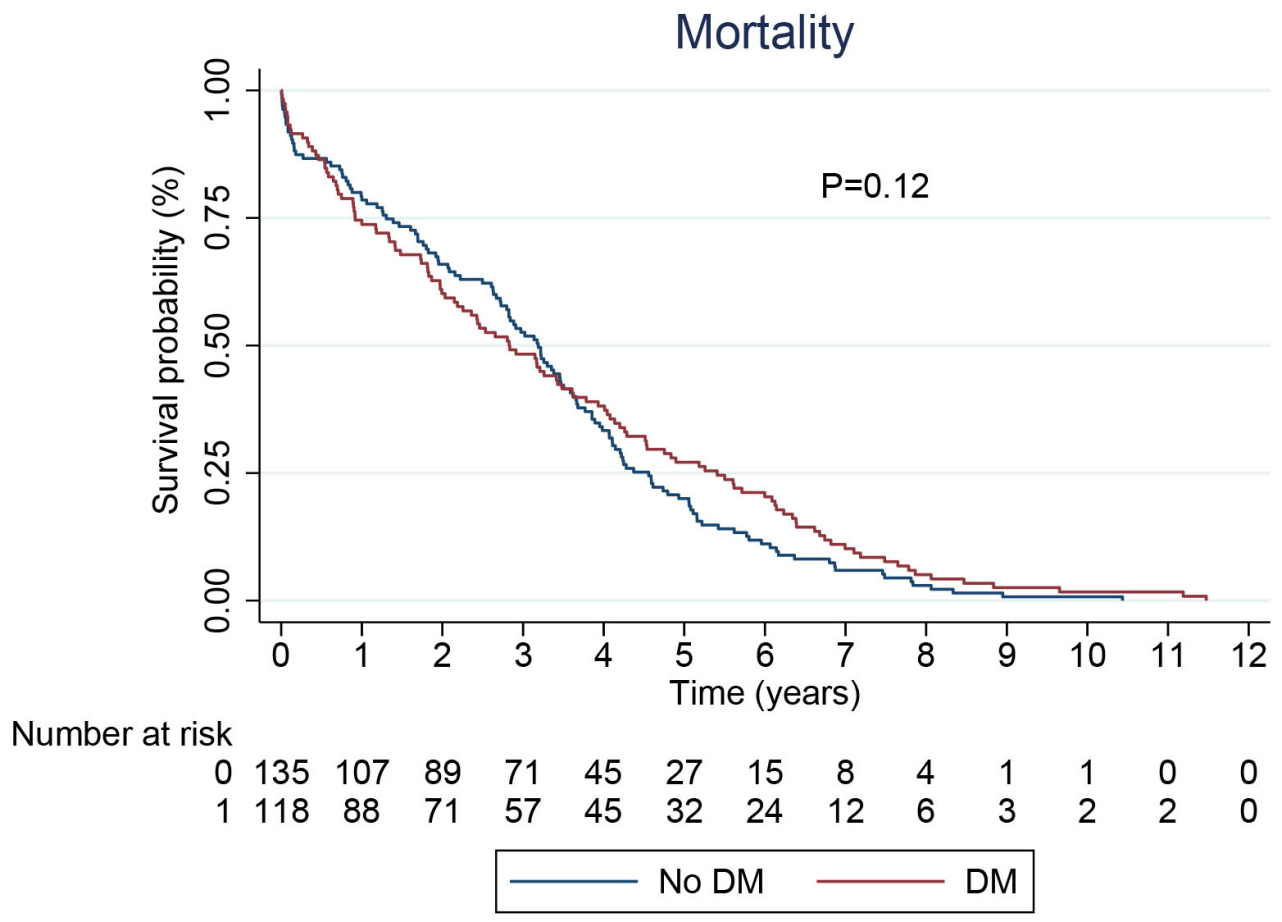
**Kaplan Meir curve based on all cause mortality for patients with and without DM, showing no difference in survival between the two groups**. DM, diabetes mellitus.

Comparing the 30-day outcome according to VARC 3 criteria, patients with and without DM had comparable stroke rates (4.8% vs. 4.1%, RR 0.9, *p* = 0.649), major or life-threatening bleeding (8.9% vs. 9.3%, RR 1.1, *p* = 0.869), myocardial infarction (1.2% vs. 2.1%, RR 1.4, *p* = 0.374), and arrhythmias (26.7% vs. 30.2%, RR 1.0, *p* = 0.657). The median length of stay was two days in both groups (*p* = 0.96). Even though the patients with DM had decreased kidney function at baseline compared with patients without DM, there was no increase in acute kidney injury or the need for dialysis in this group. Table 2 presents clinical outcomes in the study population. In Fig. 2, the *p*-value of 0.12, derived from the Kaplan-Meier curve comparing mortality rates between diabetic and non-diabetic patients, indicates that there is no statistically significant difference in survival between the two groups at the significance level of 0.05.

### 3.3 Effect of Diabetic Status and Blood Glucose Levels on Complication Rate

**Fig. 3.**
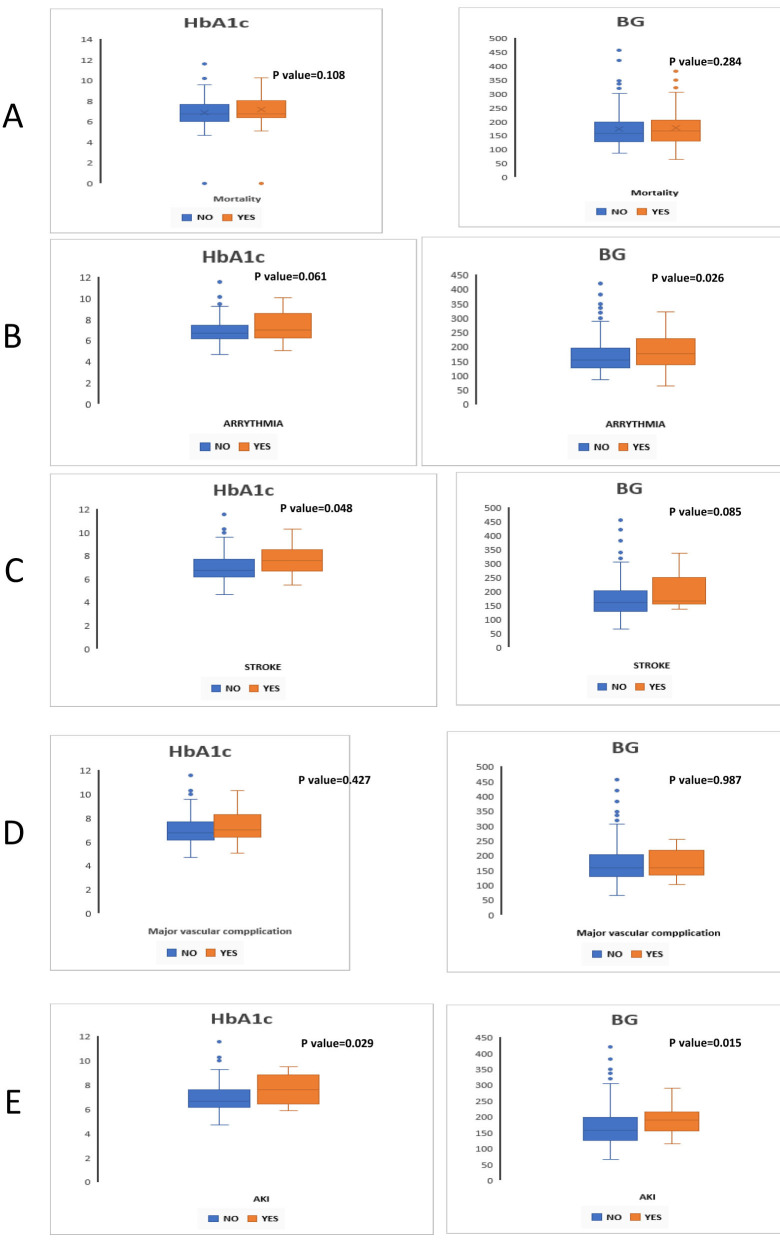
**Simple boxplot for differences between subgroups in thirty-day outcomes of diabetic patients (n = 270) after TAVR according to VARC 3 criteria and pre-procedural blood glucose (BG) and hemoglobin A1c (HbA1c) levels**. (A) Mortality, (B) Arrhythmia, (C) Stroke, (D) Major vascular complications, (E) Acute kidney injury. TAVR, transcatheter aortic valve replacement; VARC 3, Valve Academic Research Consortium 3; AKI, acute kidney injury.

In diabetic patients, 91 (33%) were uncontrolled (HbA1c >7), and 46 (17%) were well-controlled (HbA1c <5.7) (Fig. 3). We divided the DM patients into subgroups: BG <100 mg/dL (54 patients (20%)) and BG ≥126 mg/dL (170 patients (62%)). We found no differences in the baseline characteristics, basic lab tests, and procedural indexes between the subgroups (Table 3).

**Table 3.** **Baseline characteristics of diabetic patient subgroups**.

		Glucose <100	Glucose ≥126	*p* value	HbA1c <5.7	HbA1c >7	*p* value
		n = 54	n = 170		n = 46	n = 91	
Clinical characteristics							
	Age (years)	79.5 ± 10.1	80.6 ± 6.9	0.504	80.02 ± 5.89	79.15 ± 8.12	0.522
	Gender women (%)	53.3%	51.6%	0.448	72.7%	48.4%	0.147
	Body mass index (kg/m^2^)	32.1 ± 10.5	29.2 ± 4.87	0.759	28.05 ± 5.22	26 ± 4.31	0.247
	Hypertension	86.7%	92.6%	0.204	90.9%	89.0%	0.424
	Dyslipidemia	86.7%	82.6%	0.345	72.7%	84.6%	0.159
	Smoker	20%	13.7%	0.250	18.2%	18.7%	0.484
	Atrial fibrillation	26.7%	31.2%	0.357	18.2%	34.4%	0.139
	Coronary artery disease	33.3%	44.9%	0.129	45.5%	47.2%	0.457
	Peripheral vascular disease	7.1%	21.7%	0.097	27.3%	22.0%	0.346
	Past myocardial infarction	13.3%	16.8%	0.363	18.2%	19.8%	0.450
	Past stroke	7.1%	11.6%	0.304	0.0%	16.5%	0.072
	Past CABG	16.7%	10.1%	0.240	0.0%	15.2%	0.105
	STS score	7.91 ± 1.5	8.11 ± 1.4	0.365	7.97 ± 1.53	8.12 ± 1.45	0.616
Medication:							
	Insulin treatment	35.7%	27.9%	0.265	7.2%	40.4%	0.006
	Metformin	21.4%	50.3%	0.038	40.0%	42.9%	0.863
	GLP1 agonist	7.1%	6.0%	0.432	14.1%	9.0%	0.323
	SGLT2I	14.3%	5.5%	0.093	5.1%	5.7%	0.439
Laboratory							
	White blood cells (K/uL) 24 hours	8.89 ± 2.61	9.86 ± 3.77	0.494	10.94 ± 7.14	9.81 ± 3.91	0.686
	Neutrophil-absolute (K/uL) 24 hours	6.88 ± 2.19	7.97 ± 3.57	0.406	8.75 ± 6.38	8.05 ± 3.73	0.656
	Lymphocytes-absolute (K/uL) 24 hours 24 hours	0.95 ± 0.48	1.13 ± 0.66	0.402	1.31 ± 0.76	1.08 ± 0.69	0.270
	NLR 24 hours	12.5 ± 13.45	10.09 ± 10.84	0.837	8.36 ± 6.54	11.12 ± 11.41	0.583
	CRP 24 hours	39.0	7.61 ± 7.89	0.093	1.45	7.29 ± 7.19	0.172
	Creatinine (mg/dL)	1.58 ± 2.12	1.23 ± 1.04	0.740	1.66 ± 2.39	1.37 ± 1.26	0.230
Echocardiography							
	Mean LVEF (%)	54.9 ± 9.8	53.8 ± 8.3	0.264	54.5 ± 9.2	52.6 ± 10.2	0.412
	AVA (cm^2^)	0.67 ± 0.15	0.66 ± 0.17	0.815	0.67 ± 0.16	0.69 ± 0.14	0.330
	AV mean gradient (mm Hg)	48.7 ± 16.42	47.7 ± 14.45	0.526	46.3 ± 14.28	47.5 ± 15.17	0.612
Procedure-related							
	Self-expandable valve	65.1%	63.7%	0.618	67.3%	65.6%	0.762
	Post Procedural AV mean gradient (mm Hg)	6.3 ± 3.1	6.1 ± 3.5	0.721	5.9 ± 3.6	6.1 ± 3.7	0.154
Post Procedural AI							
	Minimal	78.0%	74.0%	0.361	79.0%	81.0%	0.792
	Mild	16%	20%	0.092	16%	13.7%	0.462
	Moderate	6.7%	5.6%	0.174	4.8%	5.3%	0.582
	Severe	0	0		0	0	
	Time (minutes)	73 ± 39	69 ± 41	0.267	71 ± 37	70 ± 42	0.735
	Contrast (milliliter, mL)	95 ± 45	92 ± 51	0.490	97 ± 48	95 ± 53	0.284

Values are mean ± standard deviation or n (%). *p* value considered positive <0.05. AI, aortic insufficiency; AVA, aortic valve area; AV, aortic valve; CABG, coronary artery bypass grafting; CRP, C- reactive protein; GLP1, glucagon-like peptide-1; SGLT2I, sodium-glucose cotransporter-2 Inhibitor; STS, society of thoracic surgeons; NLR, neutrophil to lymphocyte ratio; LVEF, left ventricle ejection fraction; HbA1c, hemoglobin A1c.

Two hundred and seventy of the included participants suffered from diabetes mellitus with a mean HbA1c of 7.4 ± 2.5. We measured the level of pre-procedural BG for all diabetic patients (mean 134 ± 45 mg/dL). Patients who experienced mortality had a median HbA1c of 6.9 [6.4–8.1], compared to 6.8 [6.1–7.7] for those who did not, with a *p*-value of 0.108, indicating no significant difference. Conversely, arrhythmia was associated with a higher HbA1c of 7.1 [6.3–8.6] compared to 6.7 [6.2–7.5] (*p* = 0.061), and BG levels were significantly elevated in those with arrhythmia (177 [139–228] vs. 155 [129–197], *p* = 0.026). For stroke, the median HbA1c was significantly higher at 7.6 [6.7–8.5] compared to 6.8 [6.2–7.7] (*p* = 0.048), while BG levels showed a trend without significance (165 [157–240] vs. 160 [129–203], *p* = 0.085). Major vascular complications did not show significant differences in HbA1c (7.0 [6.4–8.3] vs. 6.8 [6.2–7.7], *p* = 0.427) or BG (160 [134–218] for both groups, *p* = 0.987). However, for acute kidney injury (AKI), the median HbA1c was significantly higher at 7.6 [6.5–8.8] compared to 6.7 [6.2–7.6] (*p* = 0.029), and BG levels were also significantly higher (190 [157–210] vs. 158 [128–199], *p* = 0.015). Overall, these findings indicate that elevated HbA1c and BG levels are linked to complications such as arrhythmia, stroke, and AKI, underscoring the importance of glycemic control in patients undergoing TAVR (Fig. 3, Table 4).

**Table 4.** **Effect of diabetic status and blood glucose levels on outcome**.

	HbA1c			BG		
Complications	YES	NO	*p* value	YES	NO	*p* value
Mortality	6.9 [6.4–8.1]	6.8 [6.1–7.7]	0.108	167 [131–207]	158 [129–200]	0.284
Arrhythmia	7.1 [6.3–8.6]	6.7 [6.2–7.5]	0.061	177 [139–228]	155 [129–197]	0.026
Stroke	7.6 [6.7–8.5]	6.8 [6.2–7.7]	0.048	165 [157–240]	160 [129–203]	0.085
Major vascular complications	7 [6.4–8.3]	6.8 [6.2–7.7]	0.427	160 [134–218]	160 [129–204]	0.987
AKI	7.6 [6.5–8.8]	6.7 [6.2–7.6]	0.029	190 [157–210]	158 [128–199]	0.015

Data presented as median [IQ1–IQ3], *p* value considered positive <0.05. AKI, acute kidney injury; BG, blood glucose; HbA1c, hemoglobin A1c.

Table 5 presents the results of a multivariable logistic regression analysis examining the impact of blood glucose levels and HbA1c on outcomes such as arrhythmia, stroke, and AKI in patients with diabetes mellitus (N = 270). The analysis shows that for arrhythmia, the odds ratio for HbA1c is 1.1039 (*p* = 0.23), indicating no significant association, while blood glucose has an odds ratio of 0.998 (*p* = 0.76), also showing no significant effect. In the case of stroke, HbA1c presents an odds ratio of 1.194 (*p* = 0.36), suggesting no meaningful impact, but blood glucose is significantly associated with stroke, exhibiting an odds ratio of 1.020 (*p* = 0.013). Notably, for AKI, the odds ratio for HbA1c is 2.304 (*p* = 0.02), indicating a significant link between higher HbA1c levels and increased risk of AKI. Blood glucose levels also trend toward significance with an odds ratio of 1.0137 (*p* = 0.061).

**Table 5.** **Impact of blood glucose levels and hemoglobin A1c on outcomes (arrhythmia, stroke, AKI) in diabetes mellitus patients**.

DM patients	Arrhythmia			Stroke			AKI		
N = 270	Odds ratio	*p* value	95% confidence interval	Odds ratio	*p* value	95% confidence interval	Odds ratio	*p* value	95% confidence interval
HbA1c*	1.1039	0.23	0.9374–1.3001	1.194	0.36	0.8146–1.7503	2.304	0.02	1.0875–4.8829
BG*	0.998	0.76	0.9909–1.006	1.020	0.013	1.004–1.0375	1.0137	0.061	0.9993–1.028

*Adjusted for Gender, Age, BMI, Dyslipidemia, Smoking, History of atrial fibrillation (AFib), Peripheral vascular disease (PVD), Coronary artery disease (CAD), and Insulin treatment; *p* value considered positive <0.05; AKI, acute kidney injury; BG, blood glucose; HbA1c, hemoglobin A1c; BMI, body mass index.

## 4. Discussion

Our results suggest that (1) patients with DM have a worse cardiovascular profile and are younger than non-DM patients; (2) DM is not associated with worse outcomes at long-term follow-up after TAVR; and (3) a periprocedural glycemic state in DM can affect the major adverse cardiac events (MACE) within 30 days after TAVR, both in patients presenting with poorly controlled DM at baseline and in patients with uncontrolled blood glucose levels at the time of the procedure.

In our study, we categorized diabetic patients into controlled and uncontrolled groups based on HbA1c levels, with the threshold for uncontrolled diabetes set at ≥7%. This approach aligns with findings from many studies [13, 14, 15, 16]. For the controlled group, we conducted an extensive literature review to identify the most appropriate cutoff point.

While some studies have used 7% as a standard for both controlled and uncontrolled diabetes, we found this criterion inadequate for our research. The literature indicates that HbA1c cutoff points can vary based on factors such as age and comorbidities [11].

In our quest for an optimal cutoff, we referenced the work of Xianfeng Zhou *et al*. [12], which highlighted variations in cutoff points influenced by gender, age, and comorbidities. We found identified cut points of 5.6%, 5.8%, 5.9%, 6.0%, and 6.1% for the controlled group. Notably, our review revealed that older patients tend to have lower cutoff points. Given that the mean ± SD age of participants in our study was 81.48 ± 7.36, we deemed an HbA1c level of <5.7% as the optimal cutoff for identifying controlled diabetes in this population [11, 12].

The increasing proportion of younger patients undergoing TAVR in the last decade increased the percentage of candidates who suffer from comorbidities such as smoking, obesity, and DM [17, 18, 19]. Mortality trend estimations indicate a significant decrease in mortality. This is probably due to delivery and valve system optimization and increased provider experience, which all together decrease the trends of post-procedural acute renal failure, arrhythmia, and stroke, which is reassuring for the future of TAVR among those with diabetes.

One to one randomized controlled trials (RCT) comparing TAVR to surgical AVR in patients with diabetes are lacking. In the subgroup analysis of the PARTNER 3 study, TAVR reduced one-year mortality in patients with diabetes compared to surgical AVR [20]. In contrast, no survival advantages were reported in non-diabetes patients. In a recent case-control study that evaluated both interventions in patients with diabetes, Khan *et al*. [20] documented lower mortality but more heightened post-procedural complications in patients who underwent TAVR.

In our study, we found no differences in clinical outcomes, including mortality, between diabetic and non-diabetic patients, consistent with the studies of Tzamalis *et al*. [21] and van Nieuwkerk *et al*. [22]. However, we found an increase in mortality in the mid-term, within one year, but not in the long-term follow-up. Some earlier studies found unequivocally increased long-term but not short-term mortality in patients with diabetes [23, 24]. It is imperative to determine if this heightened mid-term mortality is due to the direct effect of diabetes on TAVR outcomes or can be solely due to the increased overall mortality risk of diabetes itself. Patients with diabetes in the general population face an increased mortality risk due to elevated rates of stroke, coronary heart disease, and other vascular diseases. It is imperative to determine if this heightened midterm mortality is due to the direct effect of diabetes on TAVR outcomes or can be solely due to the increased overall mortality risk of diabetes itself. Patients with diabetes in the general population indisputably face an increased mortality risk due to elevated rates of stroke, coronary heart disease, and other vascular diseases [25, 26, 27].

When comparing the levels of pre-procedural blood glucose and the previous glycemic state before the procedure in DM patients, we found that an uncontrolled diabetic state periprocedural can increase MACE within 30 days. Prior studies found improved kidney function in baseline renal dysfunction patients after TAVR [28, 29]. This is most probably due to the release of pressure afterload by TAVR, which plays a more important role in the recovery of type 2 chronic cardio-renal syndrome [30]. Conversely, hyperglycemia has been shown to cause kidney injury in patients with diabetes [31]. Studies have shown that raised glucose levels can reduce mesangial cells’ ability to subvert the mesangial matrix by impacting the activity of matrix metalloproteinases [32]. In addition, high glucose levels can trigger the release of cytokines and humoral mediators that can cause phenotypical and functional modifications in renal cells and tissues, impede cell growth, interact with proteins, and advance glycation end products [33, 34, 35], Eventually, this results in glomerular and tubular damage, leading to kidney disease.

Hyperglycemia causes numerous modifications in the metabolism of cardiomyocytes, including increased lipotoxic effects due to improper fatty acids utilization and elevated production of reactive oxygen species, which may lead to cardiomyocyte damage, cell death, inflammation, and fibrosis [36, 37]. These processes may all predispose the patient to conduction system disease.

Kerola *et al*. [38] show in multiple models that diabetes and hyperglycemia affect the conduction system and increase the risk of atrioventricular (AV) node block. This finding is consistent with our short term outcome data.

In our study, we acknowledge several significant limitations due to our retrospective design. One major issue is the missing data on postprocedural plasma glucose levels, which impacts the comprehensiveness of our analysis. The absence of these crucial measurements constrains our ability to fully evaluate the relationship between glycemic control and clinical outcomes following TAVR.

While we have adjusted for several confounders, including gender, age, body mass index (BMI), dyslipidemia, smoking, history of atrial fibrillation, peripheral vascular disease, coronary artery disease, and insulin treatment, it is important to note that unknown confounders may still exist.

Another study’s limitations are the potential heterogeneity due to the long enrollment period and a relatively small sample size, which may affect the reliability and generalizability of the findings.

We propose that blood glucose levels and the patient’s glycemic state prior to the procedure should be added as essential prognostic factors for assessing the pre-operative risk and selecting the appropriate treatment modality for patients with severe AS requiring TAVR. Additionally, it is crucial to conduct further research to determine whether effective diabetic control before and after TAVR could significantly improve the short- and long-term outcomes of the procedure.

## 5. Conclusions

Pre-procedural glucose levels and the patient’s previous glycemic state may offer valuable insights into the risk of major adverse events within 30 days after TAVR. To address this, we should conduct a carefully designed study involving continuous glucose monitoring. Patients with poorly controlled diabetes should undergo strict monitoring in the weeks before a planned TAVI. Hopefully, this will reduce the complication rate.

Further research is needed to better understand the underlying mechanisms causing increased glycemic variability. This could potentially lead to the development of new therapeutic approaches to prevent wide variations in peri-procedural blood glucose levels.

## Data Availability

All relevant data are included within the manuscript. All other data offered in this study are available upon proper request from the corresponding author.

## Abbreviations

TAVR, Transcatheter Aortic Valve Replacement; DM, Diabetes Mellitus; HbA1c, Hemoglobin A1c; AS, Aortic Stenosis; AKI, Acute Kidney Injury; AVR, Aortic Valve Replacement; PVD, Peripheral Vascular Disease; CAD, Coronary Artery Disease; AFib, Atrial Fibrillation; SEV, Severe Aortic Stenosis; BEV, Balloon-expandable Valve; ICU, Intensive Care Unit; VARC-3, Valve Academic Research Consortium 3; BG, Blood Glucose; SD, Standard Deviation; CI, Confidence Interval; SPSS, Statistical Package for the Social Sciences; MI, Myocardial Infarction; CABG, Coronary Artery Bypass Grafting; STS, Society of Thoracic Surgeons; LVEF, Left Ventricular Ejection Fraction; NLR, Neutrophil to Lymphocyte Ratio; K/uL, Thousand cells per microliter; mg/dL, Milligrams per deciliter; cm^2^/m^2^, Square centimeters per square meter; RR, Relative Risk; AI, Aortic Insufficiency; AVA, Aortic Valve Area; AV Gradient, Aortic Valve Gradient; CRP, C-Reactive Protein; GLP-1, Glucagon-like Peptide-1; SGLT2I, Sodium-Glucose Cotransporter-2 Inhibitor.
